# Supplementary figures and images for: A divergent cyclic nucleotide binding protein promotes Plasmodium ookinete infection of the mosquito
Source: PLoS Pathog. 2025 Sep 2;21(9):e1013467. doi: 10.1371/journal.ppat.1013467 (PMC12422582; doi:10.1371/journal.ppat.1013467)

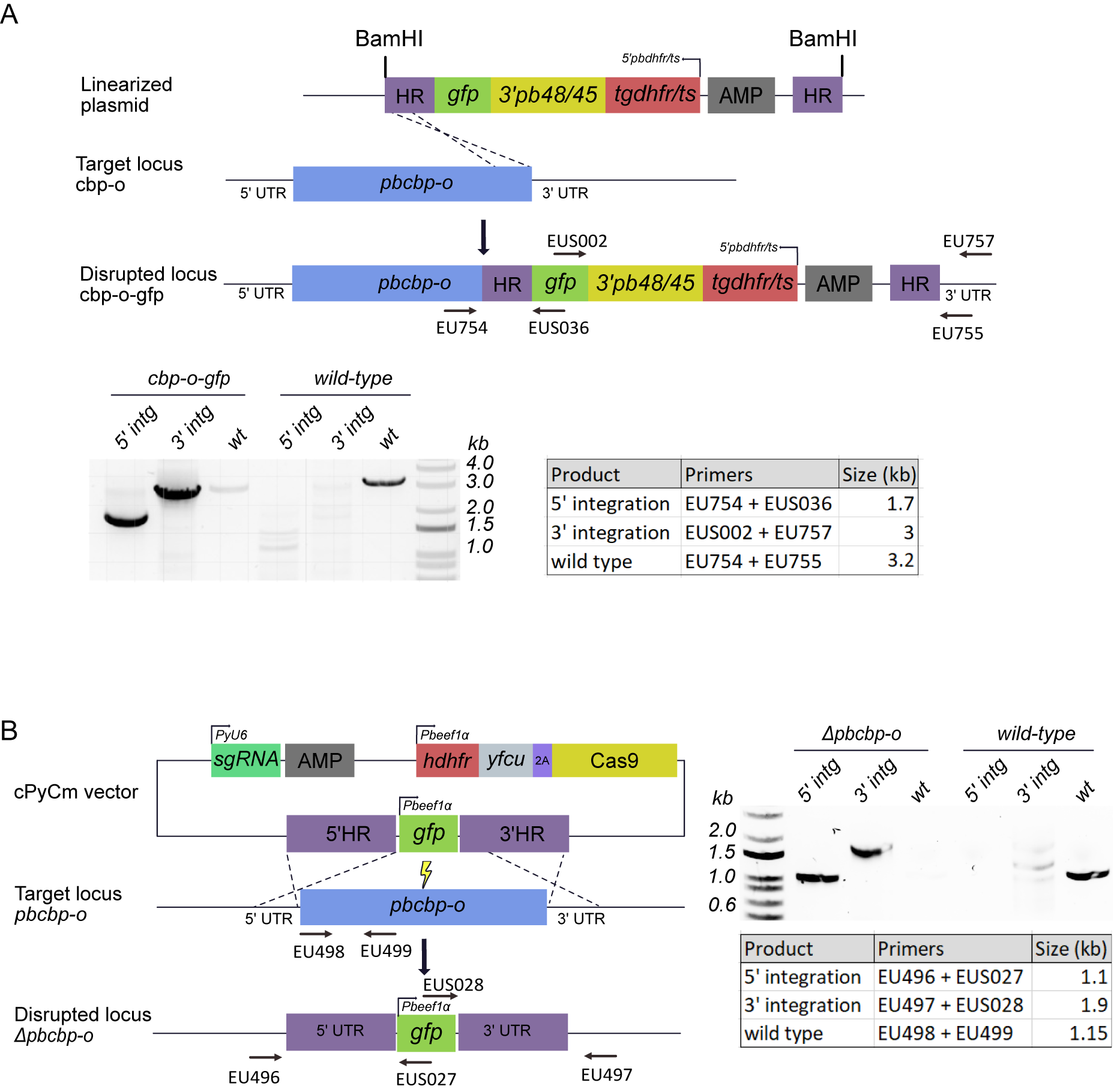

Supplement: S1 Fig — (A) CBP-O was C- terminally tagged with GFP employing a single crossover strategy where the gene was linearised using a BamHI site. (B) Full-length knock out was generated using CRISPR/Cas9 with a guide RNA mapping to the middle of the gene. The locus was replaced by a constitutively active GFP. Positions of oligonucleotides used for genotyping are indicated by arrows and identified by specific EU numbers. Genotyping by the respective diagnostic PCRs and expected sizes are indicated by agarose gel electrophoresis and a table respectively. (TIF) [file ppat.1013467.s001.tif]

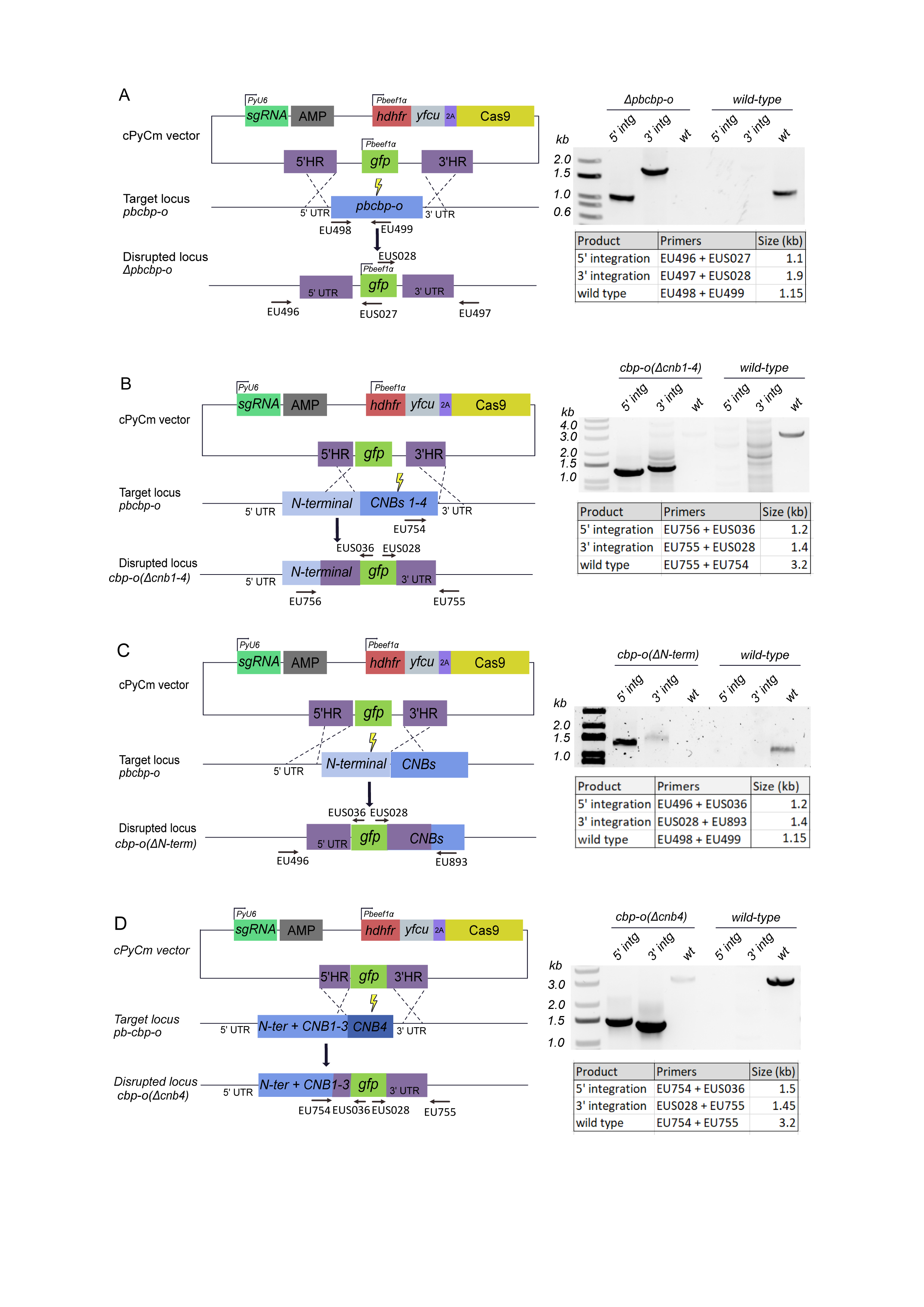

Supplement: S2 Fig — All mutant lines were created in constitutively expressing RFP line. (A) Full-length knock out (Δcbp-o) was generated using the strategy as represented in SF1A. (B) cbp-o(Δcnb1–4/x), (C) cbp-o(ΔN-term) and (D) cbp-o(Δcnb4) transgenic lines were generated by replacing the indicated loci with gfp resulting in a GFP-tagged domain mutant. Positions of oligonucleotides used for genotyping are indicated by arrows and denoted by specific EU numbers. Genotyping by diagnostic PCRs and expected sizes are indicated. (TIF) [file ppat.1013467.s002.tif]

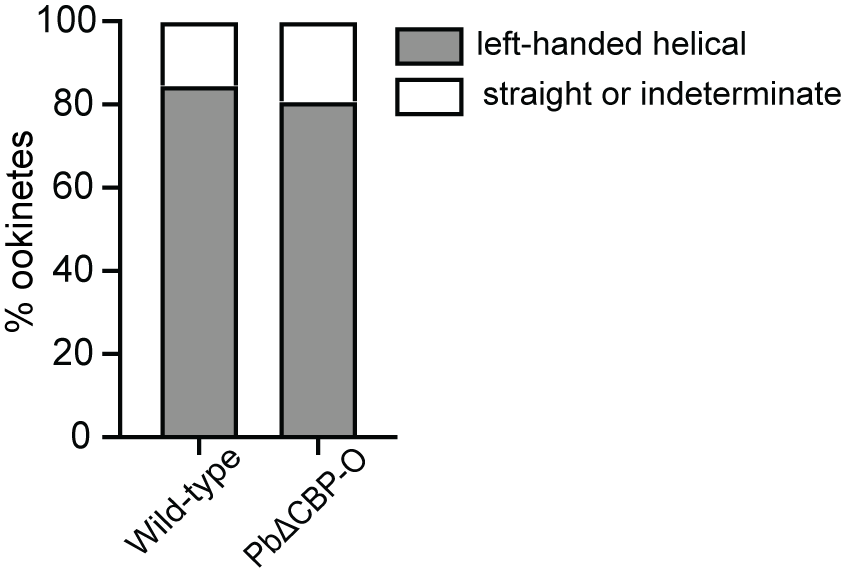

Supplement: S3 Fig — Ookinetes showing motility for at least 5 minutes were examined. n = 46 for each genotype and data is pooled from two independent replicates. (TIF) [file ppat.1013467.s003.tif]

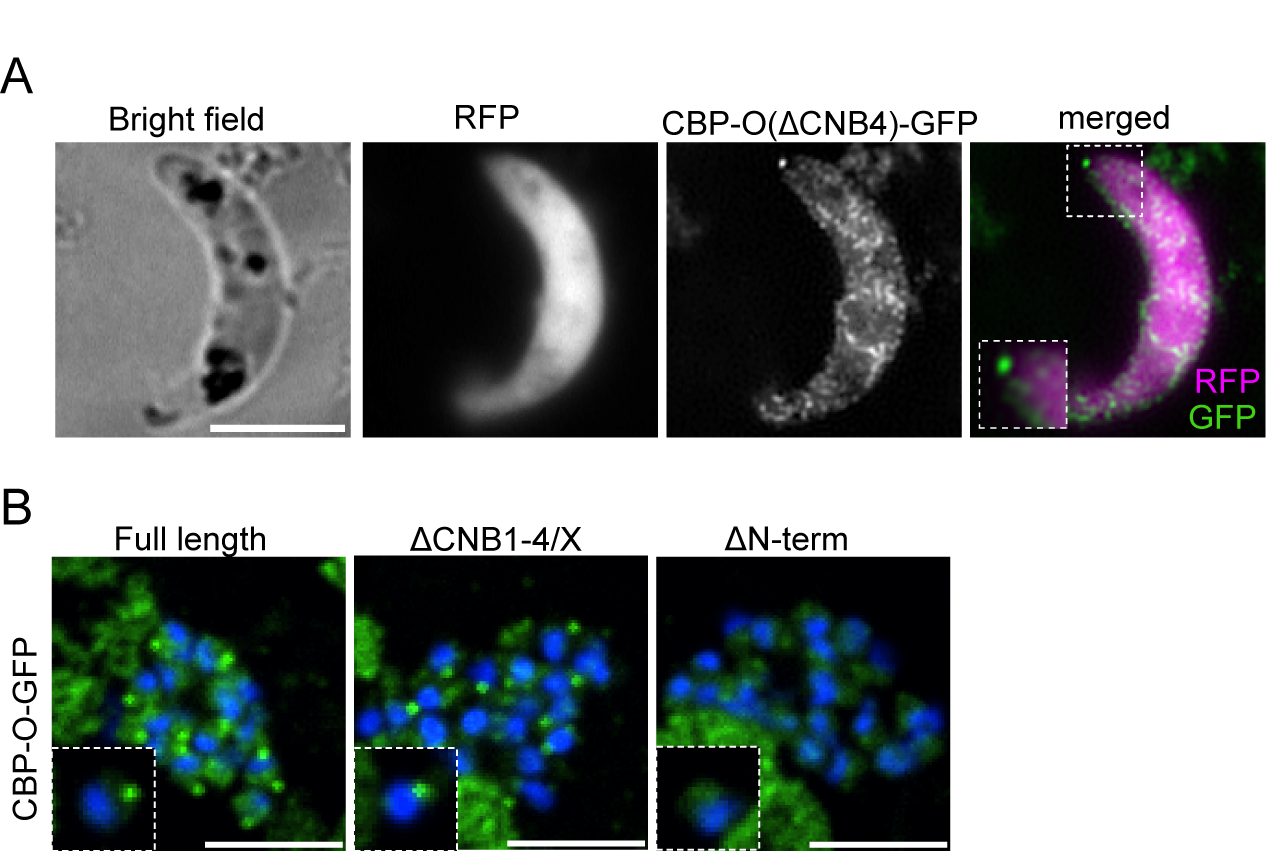

Supplement: S4 Fig — (A) Deletion of CNB4 does not impact protein localisation to the apical tip of ookinetes. Immunofluorescence microscopy of mature ookinete expressing GFP-tagged CBP-O protein lacking the terminal CNB4 domain. (B) N-terminus is required for targeting CBP-O to the merozoite tip. The full-length and CNB domain mutant protein (ΔCNB1–4/X) maintain apical localisation. Immunofluorescence microscopy of mature schizonts expressing the various domain mutant proteins. (TIF) [file ppat.1013467.s004.tif]

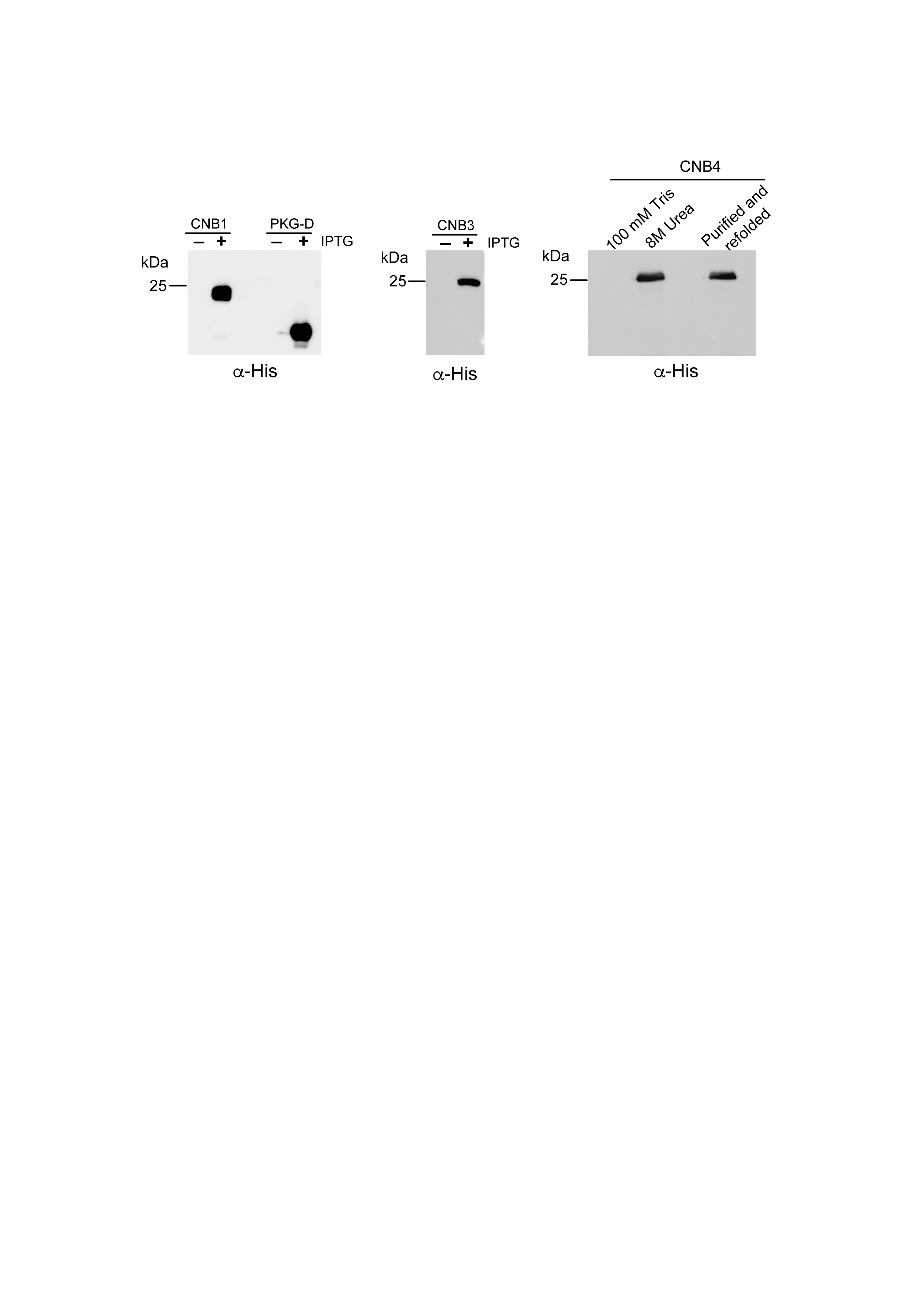

Supplement: S5 Fig — Western blot analysis of His-tagged recombinant CNB1, CNB3 and CNB4 proteins. CNB1 and 3 were isolated from solution fractions after lysis under non-denaturing conditions. CNB4 was isolated from inclusion bodies in 8M Urea buffer, followed by refolding dialysis. (TIF) [file ppat.1013467.s005.tif]
