# Supplementary material for: A divergent cyclic nucleotide binding protein promotes Plasmodium ookinete infection of the mosquito
Source: PLoS Pathog. 2025 Sep 2;21(9):e1013467. doi: 10.1371/journal.ppat.1013467 (PMC12422582; doi:10.1371/journal.ppat.1013467)
Supplement: S1 Table — (DOCX) [file ppat.1013467.s010.docx]

**S1 Table: Sequences of oligonucleotides used in the study**

**Sequences of primers used to confirm genomic integration**

| Primer | Sequence | Name |
| --- | --- | --- |
| EU496 | TTCCCTTGTCTTTGTAGCCCATAG | 5’ ∆CBP-O  5’ CBP-O(∆N-term) |
| EUS027 | GCAATTAATGTGAGTTAGCTC | 5’ ∆CBP-O |
| EU497 | GGTTACGCACCCAAAATTGTTG | 3’ ∆CBP-O |
| EUS028 | GCTGCTGGGATTACACATGG | 3’ ∆CBP-O  3’ ∆CBP-O(∆N-term)  3’ CBP-O(ΔCNB4)  3’ CBP-O(ΔCNB1-4) |
| EU498 | GGCCAAGATGTCTGCTATTTCAAAG | wt ∆CBP-O  wt ∆CBP-O(∆N-term) |
| EU499 | GTACATGCTTTCTCTTTGTTCGTTGG | wt ΔCBP-O  wt ∆CBP-O(∆N-term) |
| EU754 | TGGGTTGAAAGAAGCACACAATG | wt/ 5' CBP-O-GFP  wt CBP-O(ΔCNB1-4)  wt/ 5’ CBP-O(ΔCNB4) |
| EUS036 | GTATCTCGCAAAGCATTGAACACC | 5' CBP-GFP  5' CBP-O-(ΔCNB1-4)  5' CBP-O(∆N-term)  5’ CBP-O(ΔCNB4) |
| EUS002 | GATTAAGTTGGGTAACGCCAG | 3' CBP-O-GFP |
| EU755 | GATTAAAAGTGAGGGTTACGCACC | wt CBP-O-GFP  3'CBP-O(ΔCNB1-4)  wt/ 3’ CBP-O(ΔCNB1-4)  wt/ 3’ CBP-O(ΔCNB4) |
| EU757 | GTTACGCACCCAAAATTGTTG | 3' CBP-O-GFP |
| EU756 | GAAGGATCGGAGTGCCCATATAC | 5' CBP-O(ΔCNB1-4) |
| EUS028 | GCTGCTGGGATTACACATGG | 3' CBP-O(ΔCNB1-4)  3' CBP-O(∆N-term) |
| EU893 | TGGATATACTCTTTCCTCCATTATCCG | 3' CBP-O(∆N-term) |

**Sequences of primers for plasmid constructs**

| EU492 | aatGGTACCTTATGAAGGAATAGCAAGAG | ∆CBP-O 3'HR |
| --- | --- | --- |
| EU493 | aatGCTAGCTATAGCATGTGCTAATTCTTTAAAC | ∆CBP-O 3'HR |
| EU503 | ccgAAGCTTCAATGAAATATTTGTACGCAATG | ∆CBP-O 5'HR |
| EU504 | aatCCGCGGTTCAATATACACATATACCTTGTC | ∆CBP-O 5'HR |
| EU564 | CATACTTCGAGTTATACAATGTTATTGTTTCTAGAAATAACAAACACGTTTTAGAGCTAGAAATAGCAAGTT | ∆CBP-O gRNA |
| EU700 | CATACTTCGAGTTATACAATGTTATTGCTTCGAACAAATCAAGTTAAGTTTTAGAGCTAGAAATAGCAAGTT | CBP-O(ΔCNB1-4) gRNA |
| EU749 | CTATGACCATGATTACGCCAAGCTTGATTTAGGCATTGAGTTAAATGAAG | CBP-O(ΔCNB1-4) 5'HR |
| EU750 | CATTGAAGACCGCGGAGGTATATTATCAACAATACTCATAAATTC | CBP-O(ΔCNB1-4) 5'HR |
| EU751 | TAATATACCTCCGCGGTCTTCAATGAGTAAAG | CBP-O(ΔCNB1-4) 3'HR |
| EU752 | ATTAAATTGTAAACTTAAGGAATTCTATAGCATGTGCTAATTCTTTAAACAAG | CBP-O(ΔCNB1-4) 3'HR |
| EU708 | aatCCGCGGTCTTCAATGAGTAAAGGAGAAGAACTTTTC | CBP-O(ΔCNB1-4) GFP |
| EU709 | ccgcGGTACCTTATTTGTATAGTTCATCCATGCC | CBP-O(ΔCNB1-4) GFP |
| EU883 | catacttcgagttatacaatgttattGCATGTCTAGAATGTCCCAAAgttttagagctagaaatagcaagtt | CBP-O(∆N-term) gRNA |
| EU884 | ctatgaccatgattacgccaagcttCAATGAAATATTTGTACGCAATGC | CBP-O(∆N-term) 5'HR |
| EU885 | gctccgcggctcctttactcatattatttttataGACATTGTATAAATCGTCGTTTTTTATATC | CBP-O(∆N-term) 5'HR |
| EU886 | ggggcggccgccaatgtctataaaaataatATGAGTAAAGGAGAAGAACTTTTCAC | CBP-O(∆N-term) GFP |
| EU887 | tgagctgaagaccgcggTTTGTATAGTTCATCCATGCCATG | CBP-O(∆N-term) GFP |
| EU888 | tatacaaaccgcggtcttcaGCTCATACAAATGTTGCTATAGTCG | CBP-O(∆N-term) 3'HR |
| EU889 | gggggtaccattaaattgtaaacttaaggaattcTGACGTATTGAAACTACACCTTG | CBP-O(∆N-term) 3'HR |
| EU706 | acgTCTAGAGAACTAAACTTAAGGTTATGTGAAATG | CBP-O-GFP |
| EU707 | aatCCATGGaTGTATTAATTTTATTGTCAGGGTTTTG | CBP-O-GFP |
| EU701 | CATACTTCGAGTTATACAATGTTATTGTCGTATCCTCGGAATTTAATGTTTTAGAGCTAGAAATAGCAAGTT | CBP-O(ΔCNB4) gRNA |
| EU704 | gccgcAAGCTTAACAGTTGATGATGCTATTATAAAC | CBP-O(ΔCNB4) 5'HR |
| EU705 | aatCCGCGGTGGGACATTTTTAAGTACTTG | CBP-O(ΔCNB4) 5'HR |
| EU831 | GGAGTGCCATATGGATGCATCTATAGATTATAATAATAAAAAAAGTATC | for-PbPKG-D |
| EU832 | GATACTCGAGtcaAAATGTACCTCTCCCTATTATTC | rev-PbPKG-D |
| EU839 | CGAAGCTAGCGCTCATACAAATGTTGCTATAGTCG | for-CNB1 |
| EU840 | GCATCCTCGAGtcaCATATTCAAACGGGATATTGATATGTTTATGTC | rev-CNB1 |
| EU835 | GCAACTCATATGATACAAAAAAAAGATGGAGACTTTATAG | for-CNB3 |
| EU836 | CATACTCGAGttaATAGTTATTTTCATTACCATATATGGTTTTTAG | rev-CNB3 |
| EU837 | CGAACCTCATATGATGTTAGAAGAAAGACAACAAAATTG | for-CNB4 |
| EU838 | CCATCTCGAGttaTGTATTAATTTTATTGTCAGGGTTTTG | rev-CNB4 |

**Sequences of primers for qPCR**

| EU1022 | aatGGTACCTTATGAAGGAATAGCAAGAG | for-*Asrps7* |
| --- | --- | --- |
| EU1023 | aatGCTAGCTATAGCATGTGCTAATTCTTTAAAC | rev-*Asrps7* |
| EU1024 | ccgAAGCTTCAATGAAATATTTGTACGCAATG | for-*AsSRNP6* |
| EU1025 | aatCCGCGGTTCAATATACACATATACCTTGTC | rev-*AsSRNP6* |
