# Supplementary material for: A divergent cyclic nucleotide binding protein promotes Plasmodium ookinete infection of the mosquito
Source: PLoS Pathog. 2025 Sep 2;21(9):e1013467. doi: 10.1371/journal.ppat.1013467 (PMC12422582; doi:10.1371/journal.ppat.1013467)
Supplement: Attachment — Submitted filename: Comments.docx [file ppat.1013467.s012.docx]

Cyclic nucleotide (cGMP and cAMP) signal plays a crucial role in maintaining the invasive lifestyle of *Plasmodium*. In the *Plasmodium*, PKG and PKA are the well-known and studied cellular effectors of cyclic nucleotides. EPAC is another predicted cGMP-binding protein but remains functionally elusive. In this study, the author renamed EPAC as CBP-O (cyclic nucleotide-binding protein in ookinetes) and demonstrated that CBP-O is essential for *P. berghei* ookinetes to invade the mosquito midgut and facilitate transmission to the vector. In addition, this study reveals that PbCBP-O binds both cAMP and cGMP and expands the known repertoire of functional cyclic nucleotide effectors in *Plasmodium*. Below are some comments and suggestions:

Major comments:

> As the most critical discovery of this study, the CBP-O deficient ookinetes are normal in gliding motility but fail to develop the early oocyst in the midgut as shown in Figure 3D. There is no mechanism insight for the CBP-O function. There is lack of certainty in what exactly is causing the ookinete failure to cross the mosquito midgut epithelium. Two possibilities may explain the mutant phenotype.

1. Do the CBP-O deficient ookinetes invade the mosquito midgut epithelium? In this scenario, would injecting the CBP-O deficient ookinetes into the mosque hemocoel rescue the defect of the mutant ookinete?

2. Do the CBP-O deficient ookinetes get eliminated by the mosquito host immunity? In this scenario, would inhibiting the mosquito host immunity rescue the defect of the mutant ookinete?

These two experiments (1. mosque hemocoel injecting of ookinete, and 2. dsRNA injection for inhibiting the mosquito host immunity) are feasible (performed in many previous studies) and are necessary to figure out the defect mechanism of CBP-O deficiency.

> For the CBP-O knockout, genetic complementation experiments are necessary and would provide stronger evidence that the observed phenotypes are specifically due to CBP-O.

> A previous study (PMID: 33705380, PLOS Biology, 2021) investigated the same protein PBANKA_1025300 also in the *P. berghei* ANKA parasite. In their work, they tagged the protein with GFP at the C-terminus. live-cell imaging revealed that PBANKA_1025300 was localized exclusively to the apical region of ookinetes.

We can see a difference in the localization of GFP-tagged protein between these two studies. Please explain it. Did the author check the GFP-tagged protein product by immunoblot to confirm the successful GFP tagging?

> Line 207-218. There are many errors in the information in the description “CNB1, 3 and 4 of PbCBP-O present a threonine (Thr1852, Thr2234 and Thr3038, respectively) following the highly conserved arginine ”. It should be corrected to Thr1813, Thr2237, Thr3024.

Other errors in the “Tyr1892, Tyr2272 and Tyr3076”.

Minor concerns:

> In Figure 1B and Line 84-105, the author generated the parasite line with CBP-O tagged with RFP from the reporter parasite line (1804cl1), but it is not described in the text until Line 153-154. This information should be provided in the right place of the text to fit with Figure 1B.

> Line 111-112 and Fig 2B, the authors checked the impacts of CBP-O deficiency on parasite asexual blood stage proliferation in the mice but monitored the impacts for only three days. Extending the duration, like 7-10 days is more reasonable for a more solid conclusion.

> Line 167-168 and Fig 4F, the authors investigated the importance of three domains or regions of CBP-O, and accordingly generated three domain deletion mutants (ΔCNB4, ΔCNB1-4, and ΔN-term). Among these mutants, ΔCNB1-4 and ΔN-term ookinetes failed to cross the epithelial barrier, resulting in a significant reduction of sporozoites. The ΔCNB4 mutant exhibited no defects in oocyst load, but what about the formation of sporozoites? Did the authors also have data on sporozoite formation for ΔCNB4?

> In SF1B, the position of primer EU756 is not indicated in the schematic representation. The position of primer EU754 is also incorrect. EU754 (TGGGTTGAAAGAAGCACACAATG) was within the region of CNB, which contradicted the representation in the schematic.

> In SF2C, GFP replaces the N-terminal sequence of CBP-O, therefore it should be represented at the N-terminal of CNB in the schematic representation.

>Figure 3C: The molecular weight is not indicated in the results.

> In Figure 4D, the scale bar in the second image is different (longer) compared to those in other images. Is it normal?

> The time information for dissecting the midgut needs to be consistent. In Line 142 of the results, Infected midguts were dissected 25 hours after transmission followed by an immunofluorescence assay. In Line 376 of the material and method, Midguts were fixed and stained with P25 (plasma membrane marker) and DAPI 24 hours post-infection.
