# Supplementary material for: A divergent cyclic nucleotide binding protein promotes Plasmodium ookinete infection of the mosquito
Source: PLoS Pathog. 2025 Sep 2;21(9):e1013467. doi: 10.1371/journal.ppat.1013467 (PMC12422582; doi:10.1371/journal.ppat.1013467)
Supplement: Attachment — Submitted filename: Response_toreviewers.docx [file ppat.1013467.s013.docx]

Dear Editors,

Thank you for giving the opportunity to submit a revised version of our manuscript. We have reviewed the comments of the expert reviewers and performed additional experiments to address the comments. Please find below an itemised response each comment. All edits to manuscript are highlighted in blue text.

Sincerely,

Nisha Philip

Associate Professor in Infection
Institute of Immunology and Infection Research,
University of Edinburgh.

Editorial comments

*Editorial #1****:*** *Reviewers 2 and 3 have requested a more detailed phenotyping analysis of ookinete motility and midgut colonization. Please provide additional data and consolidate your findings to address their concerns.*

Response: We have added more information on ookinete motility including movie files with tracks and whether the ookinetes exhibit a characteristic left-handed helical motion. We have assessed the mosquito immune response and demonstrated that PbΔCBP-O ookinetes do not trigger an immune response implying the ookinetes are incapable of invading the midgut epithelium. Additionally, we have now shown that injecting ookinetes directly into the mosquito haemocoel rescues the mutant phenotype. Detailed responses are provided to specific comments.

*Editorial #2: Reviewers 1 and 3 have raised concerns regarding the cNMP binding assay. They suggest determining the relative affinities to cAMP and cGMP, and, if appropriate, including additional replicates to strengthen your results*.

Response: Our data clearly demonstrates the C-terminus and CNB3 of CBP-O bind cyclic nucleotides including cGMP and cAMP. We have shown this with both *in vitro* and *in vivo* experiments. While relative binding affinities and examining co-operative binding are important, they are beyond the scope of this study, and we are keen to investigate these aspects in follow on studies (as reviewer 1 also suggested). We have added number of replicates of the cNMP assays to the figure legend.

*Editorial #3: We would also suggest discussing your results in light of the previously published roles of cAMP signaling following host cell invasion by Plasmodium merozoites or Toxoplasma tachyzoites.*

Response: We have added the suggestion to our discussion.

**Responses to reviewers**

**Reviewer #1**

*This is a very interesting manuscript that reports on a Plasmodium berghei cyclic nucleotide-binding protein (PbCBP-O). The protein localises to the apex of ookinetes, a property that relies on the presence of its N-terminus. Disruption of the protein causes a markedly reduced ability of ookinetes to invade mosquitoes, even though ookinete gliding motility is not affected. Results were generated that are consistent with the conclusion that one of the predicted cyclic nucleotide-binding domains is able to bind to both cGMP and cAMP raising the intriguing possibility that PbCBP-O may be a mediator of both signalling pathways.
This study has been carried out carefully and the data represent an important contribution to our knowledge of cyclic nucleotide signalling in malaria parasites*.

Response: We thank the reviewer for the positive comments and considering the study an important contribution to our knowledge of cyclic nucleotide signalling in malaria parasites.

1. Reviewer #1*: Line 43/44 Consider changing to ‘gametocytes are activated and develop into gametes’
Line 48 Consider ‘mediated’ rather than ‘organised’
Line 53 Consider ‘a strong motility defect’*

Response: We have amended wording to reflect the reviewer’s suggestion.

2. *Reviewer #1: Line 94 There is a 5th recognisable (highly degenerate) CNB site in the P. falciparum orthologue (between CNB3 and CNB4) though with a lower degree of conservation than the other four CNBs. I have not checked whether this is also the case in the P. berghei orthologue, but expect that it is likely present and if so this could be added to the narrative/figure.*

Response: We thank the reviewer for sharing this information and indeed, PbCBP-O contains a fifth CNB domain (CNBX) for which we have now included description and analysis in the revised manuscript.

3. *Reviewer #1: Was salivary gland sporozoite load not determined for the CNB4 mutant (Figure 4F)? Presumably it would have been at ~WT levels*

Response: Indeed, we did determine sporozoite load for ΔCNB4 which was comparable to wild type. We have now included the data in Fig 4F

4. *Reviewer #1: An interesting finding that the N-term of the protein is required for apical localization. I certainly think that e.g. the AlphaFold models of the cAMP-binding domains of P. falciparum PKAr should be included in Figure 6B given the conclusion that PbCBP-O can bind both cNMPs.*

Response: This is a relevant suggestion and, we have now included the crystal structure of PKA-R CNB2 to the figure and our discussion.

5. Reviewer #1: Line 216, consider replacing ‘organised’ by mediated.

Response: Done.

6. *Reviewer #1: Lines 206, 216, 217 and elsewhere should specify the ‘regulatory domain of PfPKA (PKAr)’
The data shown in Figure 6C-D are consistent with the conclusion that the protein can bind both cAMP and cGMP. Please add the number of biological and technical replicates that were carried out for these experiments to the Figure 6C and D figure legends (I may have missed the information). If they are single replicates the experiments should be repeated to increase confidence in this key result.*

Response: As suggested, we have now specified PKA-R when discussing CNB domains of PKA. Experiments in Fig 6C were conducted from four independent recombinant protein preparations and 6D from three independent biological replicates. We have now included the information in the figure legend.

7. Reviewer #1: Line 268-269 This conclusion (that the protein is not an orthologue of mammalian EPAC) agrees with that drawn previously by Patel, Perrin et al 2019 regarding the P. falciparum orthologue of PbCBP-O (see Ref 9) and this should be mentioned.

Response: We apologise for not making this clear and have added the conclusions drawn by Patel, Perrin et al (Line 96-97 and 307-308).

8. *Reviewer #1: It is interesting that PbCBP-O is localised conveniently close to GCβ. Could you get sufficient ookinete material for pull down experiments to explore potential binding partners in future work beyond the current manuscript?*

Response: This is certainly very interesting and, we attempted several IP experiments using the GFP tagged CBP-O. However, we were unsuccessful in isolating sufficient material for Mass spectrometry. We are optimistic that future work using Bio/Turbo ID could discover potential binding partners.

8. *Reviewer #1: It would definitely be good to explore in future work the suggestion that cAMP may interact with PbCBP-O during transit of the epithelial later since ACβ and PKAr are expressed in mature ookinetes. Is there any expression of PKAc in mature ookinetes?? It is intriguing that this protein may be a mediator of both cGMP and cAMP signalling in ookinetes (and perhaps blood stages).*

Response: These are all interesting points and questions that merit pursuing. PKA-C protein was detected in the proteomic studies of ookinetes reported in PMID: 24594931 and we have now highlighted that in our discussion.

**Reviewer #2**

Cyclic nucleotide (cGMP and cAMP) signal plays a crucial role in maintaining the invasive lifestyle of *Plasmodium*. In the *Plasmodium*, PKG and PKA are the well-known and studied cellular effectors of cyclic nucleotides. EPAC is another predicted cGMP-binding protein but remains functionally elusive. In this study, the author renamed EPAC as CBP-O (cyclic nucleotide-binding protein in ookinetes) and demonstrated that CBP-O is essential for *P. berghei* ookinetes to invade the mosquito midgut and facilitate transmission to the vector. In addition, this study reveals that PbCBP-O binds both cAMP and cGMP and expands the known repertoire of functional cyclic nucleotide effectors in *Plasmodium*. Below are some comments and suggestions:

Response: We thank the reviewer for suggestions to strengthen the manuscript.

**Major concerns**

1. *Reviewer #2:*  *As the most critical discovery of this study, the CBP-O deficient ookinetes are normal in gliding motility but fail to develop the early oocyst in the midgut as shown in Figure 3D. There is no mechanism insight for the CBP-O function. There is lack of certainty in what exactly is causing the ookinete failure to cross the mosquito midgut epithelium. Two possibilities may explain the mutant phenotype.*

*1. Do the CBP-O deficient ookinetes invade the mosquito midgut epithelium? In this scenario, would injecting the CBP-O deficient ookinetes into the mosque hemocoel rescue the defect of the mutant ookinete?*

*2. Do the CBP-O deficient ookinetes get eliminated by the mosquito host immunity? In this scenario, would inhibiting the mosquito host immunity rescue the defect of the mutant ookinete?*

*These two experiments (1. mosque hemocoel injecting of ookinete, and 2. dsRNA injection for inhibiting the mosquito host immunity) are feasible (performed in many previous studies) and are necessary to figure out the defect mechanism of CBP-O deficiency.*

Response: We have now conducted further experiments to determine if CBP-O deficient ookinetes are incapable of invading the midgut epithelium and, therefore cannot continue development or the mosquito immune response eliminates the parasites. Haemocoel injections restored the mutant phenotype. Comparable sporozoite numbers were recovered from salivary glands from wild type and mutant parasites demonstrating that when the epithelial barrier is removed, PbΔCBP-O ookinetes can successfully develop into salivary gland sporozoites (Fig 3G). We also assessed the mosquito immune response by examining mRNA levels of *Anopheles stephensi* SRPN6, an early molecular marker of ookinete invasion and demonstrated that PbΔCBP-O fail to induce similar levels SRPN6 expression as wildtype (Fig. 3F) This suggests the mosquito immune response is not triggered by PbΔCBP-O ookinetes implying the mutant ookinetes do not breach the midgut epithelium.

2. *Reviewer #2 For the CBP-O knockout, genetic complementation experiments are necessary and would provide stronger evidence that the observed phenotypes are specifically due to CBP-O*

Response: PBANKA_1025300 is a 10kb gene and we have deleted the complete ORF to generate our knockout line. Complementing the transgenic line with plasmids containing large repair regions is extremely challenging without introducing errors in nucleotide sequences during DNA amplification and cloning from a high AT rich genome. To strengthen our evidence, we have assessed the CBP-O deficient phenotype in two independently generated transgenic lines. Moreover, our numerous domain deletion mutants showing distinct protein localisation and phenotypes provide strong evidence that the phenotypes are specific to CBP-O.

3. *Reviewer #2: A previous study (PMID: 33705380, PLOS Biology, 2021) investigated the same protein PBANKA_1025300 also in the P. berghei ANKA parasite. In their work, they tagged the protein with GFP at the C-terminus. live-cell imaging revealed that PBANKA_1025300 was localized exclusively to the apical region of ookinetes. We can see a difference in the localization of* *GFP-tagged protein between these two studies. Please explain it. Did the author check the GFP-tagged protein product by immunoblot to confirm the successful GFP tagging?*

Response: Indeed, the previous study demonstrated that PBANKA_1025300 was localised to the apical region of merozoites, ookinetes and sporozoites and we have referred to this observation in our manuscript. The slight variation in localisation of the protein could be explained either due to different processing techniques or that under our experimental conditions (including live cell imaging on a high-resolution Nikon Spinning disk microscope), we could have captured conoid extrusion/protusion of the ookinete. Notably, this is analogous to the localisation of the *T. gondi* orthologue when the tachyzoites are extracellular. We have added this consideration in the revised manuscript.

Separation and transfer of proteins > 300kDa by western blotting is very challenging and often unfeasible. PbCBP-O-GFP has a predicted molecular weight of ~ 400kDa and we cannot detect the protein by western blotting. However, integration PCRs demonstrating C-terminal GFP tagging of CBP-O and the specific localisation of GFP to the ookinete tip indicate the fluorescence we observe is indeed due to PbCBP-O-GFP.

4. *Reviewer #2:* Line 207-218. There are many errors in the information in the description “CNB1, 3 and 4 of PbCBP-O present a threonine (Thr1852, Thr2234 and Thr3038, respectively) following the highly conserved arginine ”. It should be corrected to Thr1813, Thr2237, Thr3024.

Other errors in the “Tyr1892, Tyr2272 and Tyr3076”.

Response: We applaud the reviewer’s attention to detail and thank them for spotting the numbering errors in amino acids. They have now been corrected.

**Minor concerns**

1. *Reviewer #2: In Figure 1B and Line 84-105, the author generated the parasite line with CBP-O tagged with RFP from the reporter parasite line (1804cl1), but it is not described in the text until Line 153-154. This information should be provided in the right place of the text to fit with Figure 1B.*

Response: We have brought this information forward (Line106) and, also moved the schematic of the transgenic line to S1A Fig.

2. *Reviewer #2: Line 111-112 and Fig 2B, the authors checked the impacts of CBP-O deficiency on parasite asexual blood stage proliferation in the mice but monitored the impacts for only three days. Extending the duration, like 7-10 days is more reasonable for a more solid conclusion.*

Response: In our experience, three replication cycles are reasonable to detect proliferation defects during asexual stages unless the effect is very subtle. For clarity we have now added in the text that proliferation was monitored for three replication cycles.

3. *Reviewer #2:*  *Line 167-168 and Fig 4F, the authors investigated the importance of three domains or regions of CBP-O, and accordingly generated three domain deletion mutants (ΔCNB4, ΔCNB1-4, and ΔN-term). Among these mutants, ΔCNB1-4 and ΔN-term ookinetes failed to cross the epithelial barrier, resulting in a significant reduction of sporozoites. The ΔCNB4 mutant exhibited no defects in oocyst load, but what about the formation of sporozoites? Did the authors also have data on sporozoite formation for ΔCNB4?*

Response: Indeed, we have sporozoite counts ΔCNB4 which showed comparable levels to wild-type and we have included the data in Fig 4F.

4. *Reviewer #2: In SF1B, the position of primer EU756 is not indicated in the schematic representation. The position of primer EU754 is also incorrect. EU754 (TGGGTTGAAAGAAGCACACAATG) was within the region of CNB, which contradicted the representation in the schematic.*

*In SF2C, GFP replaces the N-terminal sequence of CBP-O, therefore it should be represented at the N-terminal of CNB in the schematic representation.*

Response: We appreciate the reviewer for noticing the imprecise schematic of generation of GFP-ΔCNB line and absence of EU756 primer position. We have now updated all schematics to highlight and clarify what regions of the genome are replaced including location of primers.

5. *Reviewer #2: Figure 3C: The molecular weight is not indicated in the results*.

Response: The molecular weights are now included.

6. *Reviewer #2: In Figure 4D, the scale bar in the second image is different (longer) compared to those in other images. Is it normal?*

Response: The second image was at a slightly lower magnification and hence the longer scale bar. To harmonise the figure, we have now replaced the panel with an image of identical magnification.

7. *Reviewer #2* *The time information for dissecting the midgut needs to be consistent. In Line 142 of the results, Infected midguts were dissected 25 hours after transmission followed by an immunofluorescence assay. In Line 376 of the material and method, Midguts were fixed and stained with P25 (plasma membrane marker) and DAPI 24 hours post-infection.*

Response: We have amended the information to 24-25 hours which appropriately reflects the timeline for the experiments.

**Reviewer #3**

*Cyclic nucleotide signaling plays an essential role in several processes at multiple stages of Plasmodium. The parasite encodes 3 putative cNMP binding proteins, of which two (PKA and PKG) are well studied. There is a third protein with 4 predicted cNMP binding sites and a long N-terminal extension whose role is less well studied. It is known to be dispensable in the asexual erythrocytic stages.

The manuscript by Kwecka et al aims to fill this gap. They describe the essential functions of this protein, PbCBP-O in invasion by P. berhgei ookinetes of the midgut epithelium. Using parasites with GFP-tagged protein, they determine that it localizes to the apical end of ookinetes. They further characterize the protein by determining that 3 of 4 cNMP binding sites are essential for this function, while the amino end is required for proper localization to the apical end. Studies using recombinant individual cNMP binding sites were conducted to determine that each one is capable of binding both cAMP and cGMP. Using gene replacement, they demonstrate that parasites lacking the protein form ookinetes normally and these ookinetes appear to have normal motility and secretion of micronemal proteins. Intriguingly, they do not form oocysts. These results are interpreted to mean that ookinetes are unable to invade the midgut epithelium. The molecular genetic studies are well done and the manuscript is written clearly. The biochemical characterization and data on ookinete motility needs strengthening along with the assertion that CBP-O is an effector protein.*

1. *Reviewer #3 Ookinete motility has various parameters – speed, displacement and chirality doi: 10.1111/cmi.12283. The authors should measure displacement and chirality*.

Response: We thank the reviewer for the above suggestions. Majority of our ookinetes exhibit a meandering forward movement and although we use Matrigel in our motility assays, the videos are captured in 2D and therefore we cannot accurately determine the percentage of turns observed in a track as reported in doi: 10.1111/cmi.12283. However, we assessed the chirality of ookinete movement and it is clear from our assays that majority of the wild type and mutant ookinetes (~80%) exhibit a left-handed helical motion (S3 Fig). We have included representative videos in the supplementary information.

2. *Reviewer #3: Authors conclude that the lack of P25 staining implies lack of ookinete invasion. Alternative scenarios such as dysmorphic OES, increased ookinete nitrosylation, or clearance during epithelial migration should be examined. It would be advisable to use orthogonal P25-independent approaches to detect oocysts. Epithelial invasion by ookinetes causes upregulation of mosquito markers - their assessment can provide a direct assessment of ookinete invasive ability.*

Response: P25 antibody labels the surface of ookinetes and oocysts, and staining can be confidently used to detect ookinetes/oocysts in the midgut epithelium (PMID: 31969456, PMID: 15456781). We agree with the reviewer that assessment of mosquito immune markers triggered in response to ookinete invasion will provide further evidence. We have now examined mRNA levels of *Anopheles stephensi* SRPN6, a molecular marker of ookinete invasion and demonstrated that PbΔCBP-O fail to induce similar levels SRPN6 expression as wildtype (Fig 3F).

3. *Fig 4D: The text claims that PbCBP-O binds cAMP and cGMP in vivo but results in Fig 4D do not support the assertion. It should include Pb-CBP-GFP parasites as well to provide context for the efficiency of cGMP binding in the deltaN mutant.*

Response: Separation and transfer of proteins > 300kDa by western blotting is very challenging and often unfeasible. PbCBP-GFP has a predicted molecular weight of ~ 400kDa and we cannot detect the protein by western blotting. However, we could successfully detect ΔN-term-GFP (196 kDa) and ΔCNB-GFP (233kDa) proteins and utilised them for the cNMP assays. We have reworded the text to assert that the C-term of CBP-O binds cAMP and cGMP *in vivo* (Line 291)*.*

4. *Reviewer #3: Figure 6: Knowledge of the binding affinities of each CNB site for cGMP and cAMP would be more relevant to determining the potential cNMP binding properties of the protein. Further, the sites may show cooperative binding as seen in PKG. It is surprising that CNB4, the most conserved site, did not show any binding, despite model predictions. Authors speculate that this results from improper folding. Have they tested different fusion proteins to improve solubility or CD studies to determine proper refolding?*

Response: While relative binding affinities of each CNB site for cAMP or cGMP and possibility of cooperative binding would be highly relevant and appealing for mechanistic understanding of CBP-O function, detailed biochemical and structural studies are beyond the scope of this study, but we plan to pursue them as follow on investigations. We attempted GST fusion for CNB4 and were unable to improve solubility. Nevertheless, we have clearly shown than PbCNB3 *in vitro,* and the C-terminus (containing all putative CNB domains) *in vivo* are capable of binding cAMP and cGMP.

5. *Reviewer #3: The authors have the means to provide a more informative description of protein expression that is not limited to ookinetes. Line 286 states that the protein is expressed at the tip of merozoites but I did not find these data in the main text or in Supplementary information. Additionally, what is the protein’s expression and localization in sporozoites, the other motile and invasive stage in mosquitoes?*

Response: A large-scale study of *T. gondi* and *P. berghei* apical complex proteins (PMID: 33705380) demonstrated the expression of PBANKA_1025300 (or CBP-O here) and the *T. gondi* orthologue at the apical tip of merozoites, ookinetes, sporozoites and tachyzoites which we referred to in our introduction. We have now included data in S4B Fig demonstrating the apical expression of CBP-O in merozoites and more importantly, the data reveals that N-terminus of the protein is required for protein localisation to the merozoite apex.

6. *Reviewer #3: Discussion Lines 260-261 states that “cyclic nucleotide signaling through CBP-O is critical for P. berghei ookinetes to invade…”. I would urge caution in claiming that there is signaling through CBP-O or that CBP-O is an effector protein. Data presented in this manuscript, in my opinion, do not show an effect on signaling. There is a clear phenotype for sure, but is this due to impaired signaling? As noted by the authors, CBP-O does not appear to encode any identifiable domain except of CBPs, so is it a sensor or an effector*?

*Along the lines of the previous comment, the Discussion could include a Discussion of CBP-O’s potential role in sequestering cyclic nucleotides either to increase their local concentration or to prevent access by effector kinases*

Response: Cyclic nucleotides (both cAMP and cGMP) are widely considered second messengers and therefore it is reasonable to assume that cyclic nucleotide binding proteins will influence signalling pathways either directly (through activity) or indirectly (sequestering or storing cAMP/or cGMP as the reviewer suggests). We have reworded the sentence and have included the possibility of sequestration in the discussion where we had initially noted the proximity of GCβ to CBP-O.
